# Critical role of phosphorylation of serine 165 of YBX1 on the activation of NF-κB in colon cancer

**DOI:** 10.18632/oncotarget.5120

**Published:** 2015-08-08

**Authors:** Lakshmi Prabhu, Rasika Mundade, Benlian Wang, Han Wei, Antja-Voy Hartley, Matthew Martin, Kyle McElyea, Constance J. Temm, George Sandusky, Yunlong Liu, Tao Lu

**Affiliations:** ^1^ Department of Pharmacology and Toxicology, Indiana University School of Medicine, Indianapolis, IN, USA; ^2^ Center for Proteomics and Bioinformatics, Case Western Reserve University, Cleveland, Ohio, USA; ^3^ Department of Pathology and Laboratory Medicine, Indiana University School of Medicine, Indianapolis, IN, USA; ^4^ Department of Medical and Molecular Genetics, Medical Research and Library Building, Indianapolis, IN, USA; ^5^ Center for Computational Biology and Bioinformatics, Health Information and Translational Sciences, Indianapolis, IN, USA; ^6^ Department of Biochemistry and Molecular Biology, Indiana University School of Medicine, Indianapolis, IN, USA

**Keywords:** colon cancer, NF-κB, phosphorylation, serine, YBX1

## Abstract

Y-box binding protein 1 [YBX1] is a multifunctional protein known to facilitate many of the hallmarks of cancer. Elevated levels of YBX1 protein are highly correlated with cancer progression, making it an excellent marker in cancer. The connection between YBX1 and the important nuclear factor κB [NF-κB] has never been reported. Here, we show that overexpression of wild type YBX1 [WT-YBX1] activates NF-κB, suggesting that YBX1 is a potential NF-κB activator. Furthermore, using mass spectrometry analysis we identified novel phosphorylation of serine 165 [S165] on YBX1. Overexpression of the S165A-YBX1 mutant in either HEK293 cells or colon cancer HT29 cells showed dramatically reduced NF-κB activating ability as compared with that of WT-YBX1, confirming that S165 phosphorylation is critical for the activation of NF-κB by YBX1. We also show that expression of the S165A-YBX1 mutant dramatically decreased the expression of NF-κB-inducible genes, reduced cell growth, and compromised tumorigenic ability as compared with WT-YBX1. Taken together, we provide the first evidence that YBX1 functions as a tumor promoter via NF-κB activation, and phosphorylation of S165 of YBX1 is critical for this function. Therefore, our important discovery may lead to blocking S165 phosphorylation as a potential therapeutic strategy to treat colon cancer.

## INTRODUCTION

Y-box binding protein [YBX1], also known as nuclease-sensitive element-binding protein [NSEP1] or DNA-binding protein B [DBPB] is encoded by the *YBX1* gene. YBX1 contains a highly conserved “cold-shock” domain [CSD] and is a member of the CSD superfamily. YBX1 is a multifunctional DNA/RNA-binding protein that regulates transcription and translation. The CSD of YBX1 specifically interacts with DNA and RNA and regulates many DNA- and mRNA-dependent processes, including DNA transcription, replication, repair, environmental stress, chromatin remodeling, as well as pre-mRNA splicing, *etc.* [1].

High expression of YBX1 is frequently detected in a wide variety of cancers and closely relates to the progression and poor prognosis of these cancers. Elevated levels of YBX1 are seen in melanoma, osteosarcomas, prostate, breast, squamous cell, lung, ovarian, thyroid, and colorectal [CRC] cancers [2, 3]. Shibao K *et al* [4] first demonstrated that YBX1 expression is elevated in CRC and positively correlates with DNA topoisomerase II α and proliferating cell nuclear antigen [PCNA] expression but not with multi-drug resistance gene [MDR1]. Later, Vaiman *et al* [5] showed that in colon cancer cells, YBX1 accumulates in the nuclei in response to the chemotherapy drug vinblastine and is associated with development of vinblastine resistance and elevated expression of P-MDR1. YBX1 promotes tumorigenesis, cell proliferation, replicative immortality, angiogenesis, invasion, and metastasis, most of which are the ‘hallmarks of cancer’ proposed by Hanahan and Weinberg [6, 7]. Furthermore, Lee C *et al* [8] showed that, YBX1 protein, when knocked down using RNAi, reduces tumor growth in human epidermal growth factor receptor [HER-2] positive breast cancer cells, confirming that YBX1 functions as a tumor promoter in breast cancer. It is now widely accepted that YBX1 is an oncogene. It has been previously demonstrated that the phosphatidylinositide 3-kinase [PI3K/AKT] pathway causes the phosphorylation of S102 on YBX1 protein and governs its nuclear translocation in breast cancer cells [9, 10]. When this site is disrupted, YBX1 is unable to translocate to the nucleus and activate the target genes, leading to a reduction in tumor growth in human breast cancer cells [10–12].

NF-κB is a family of transcription factors that regulates the expression of genes involved in inflammation, cell proliferation, differentiation, and survival of immune responses [13]. Constitutively active NF-κB has been found in multiple types of cancer [14, 15]. There are five proteins in the mammalian NF-κB family: RelA [p65], RelB, c-Rel, p50/p105 and p52/p100. All proteins in the NF-κB family share a Rel homology domain [RHD] in their N-terminus, which results in their classification as NF-κB/Rel proteins. RHD is essential for dimerization as well as for binding to cognate DNA elements. The prototypic NF-κB is the heterodimer of p65 and p50. The activity of NF-κB is primarily regulated by interaction with inhibitory IκB [inhibitor of NF-κB] proteins. In most cells, NF-κB is present as a latent and inactive IκB-bound complex in the cytoplasm [16]. When a cell receives any of a multitude of extracellular signals such as stress, cytokines, free radicals, radiation etc., NF-κB rapidly enters the nucleus and activates target gene expression [17]. The molecular identification of p65 subunit as a member of the reticuloendotheliosis (REL) family provided the first evidence that linked NF-κB to cancer, as v-REL is an oncoprotein of the REL retrovirus (REV-T) [18]. NF-κB therefore holds great potential for use as a therapeutic target.

Although there has been increasing evidence that YBX1 and NF-κB could co-regulate different families of genes like cyclin, TGF-β *etc* [www.evexdb.org], to date it is unknown if YBX1 can activate NF-κB directly. In the study described here, we found that overexpression of WT-YBX1 could activate NF-κB. This is the first evidence suggesting that YBX1 is an activator of NF-κB. Using mass spectrometry analysis, we identified phosphorylation of the novel S165 site on YBX1. Overexpression of S165A-YBX1 mutant led to decreased NF-κB inducible gene expression, as well as reduced cell growth and tumorigenic ability as compared with the effect of the overexpression of WT-YBX1. We proposed that phosphorylation of S165 on YBX1 is essential for the biological function of YBX1. Thus, we provided a novel mechanism for the regulation of YBX1, which can be used as a potential strategy to control YBX1 and further lead to down-regulation of the activity of NF-κB in cancer.

## RESULTS

### Identification of novel phosphorylation modification on S165 of YBX1

**Figure 1:**
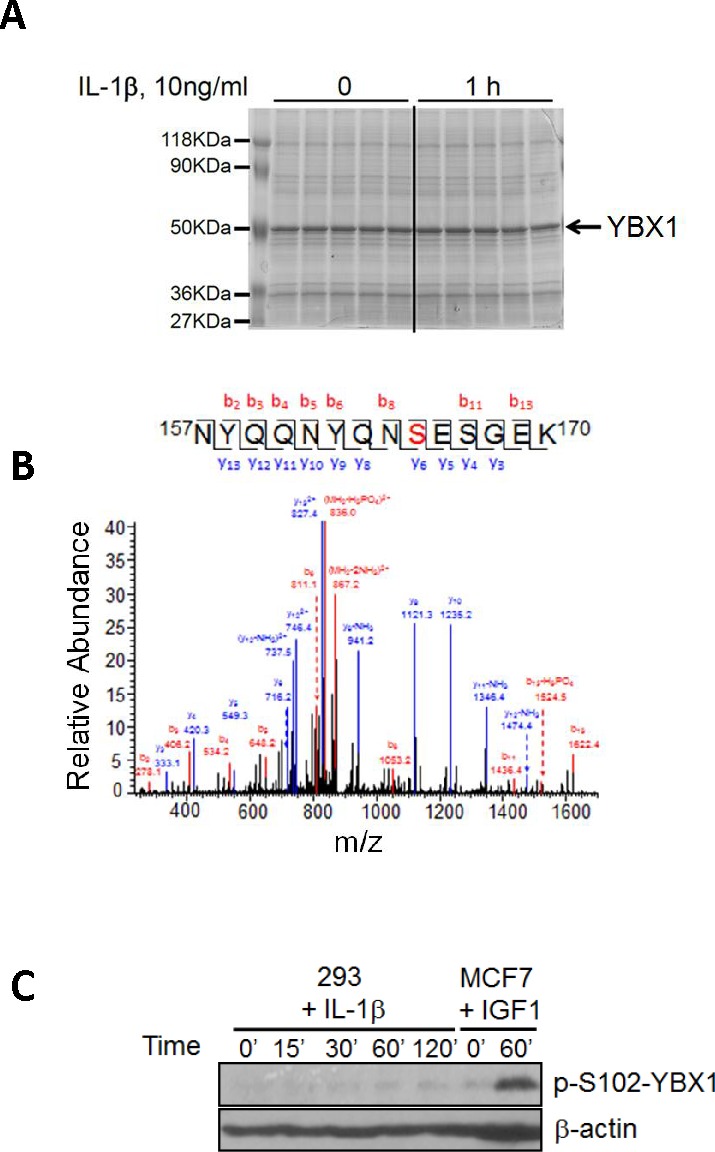
Identification of phosphorylation of S165 on YBX1 **A.** SDS-PAGE gel stained with GelCode blue, showing a strong Flag-tagged YBX1 band that was pulled down using anti-Flag antibody via co-immunoprecipitation. **B.** Mass Spectrometry data for YBX1, showing that in response to IL-1β treatment, serine 165 (S165) is phosphorylated. A mass shift of 80Da was observed, indicating the existence of phosphorylation modification. **C.** Western blot, showing that S102 is not phosphorylated in 293 cells upon IL-1β stimulation. In contrast, S102 is phosphorylated in breast cancer MCF7 cells upon IGF-1 treatment.

To date, YBX1 has been shown to be phosphorylated only on S102 after insulin growth factor 1 (IGF-1) treatment [10], an event that fundamentally affects YBX1 function in human breast cancer MCF7 cells [10, 11]. Since it is very common that the same protein can be phosphorylated on different amino acids, such as serine, threonine [T], and tyrosine [Y], or on different residues in response to different stimuli or in different cell systems, we wondered whether treating 293 cells with well-known NF-κB activating cytokine IL-1β would lead to differential phosphorylation of YBX1. In order to screen for phosphorylation sites on YBX1, 293 cells with stable expression of Flag-tagged WT-YBX1 were treated with IL-1β for 1h, and anti-Flag-antibody was used to pull down Flag-WT-YBX1. As shown in Figure 1A, a strong YBX1 band was obtained in a GelCode blue stained gel. This band was excised and further analyzed by MS assay, revealing that YBX1 is phosphorylated on S165 residue upon the treatment with IL-1β. In contrast, no S102 phosphorylation was observed in this sample (Figure 1B). We then treated 293 cells with IL-1β for different times, and treated breast cancer MCF7 cells with IGF-1 for 1h. Western assay (Figure 1C) further confirmed that in response to IL-1β, no S102 phosphorylation was induced in IL-1β treated 293 cells, while a strong pS102 signal was observed in IGF1 treated MCF7 cells. This interesting finding confirmed the differential phosphorylation of distinct sites of YBX1 in separate experimental systems, leading us to further investigate the significance of S165 phosphorylation on the function of YBX1.

### S165 phosphorylation of YBX1 is essential for the activation of NF-κB

**Figure 2:**
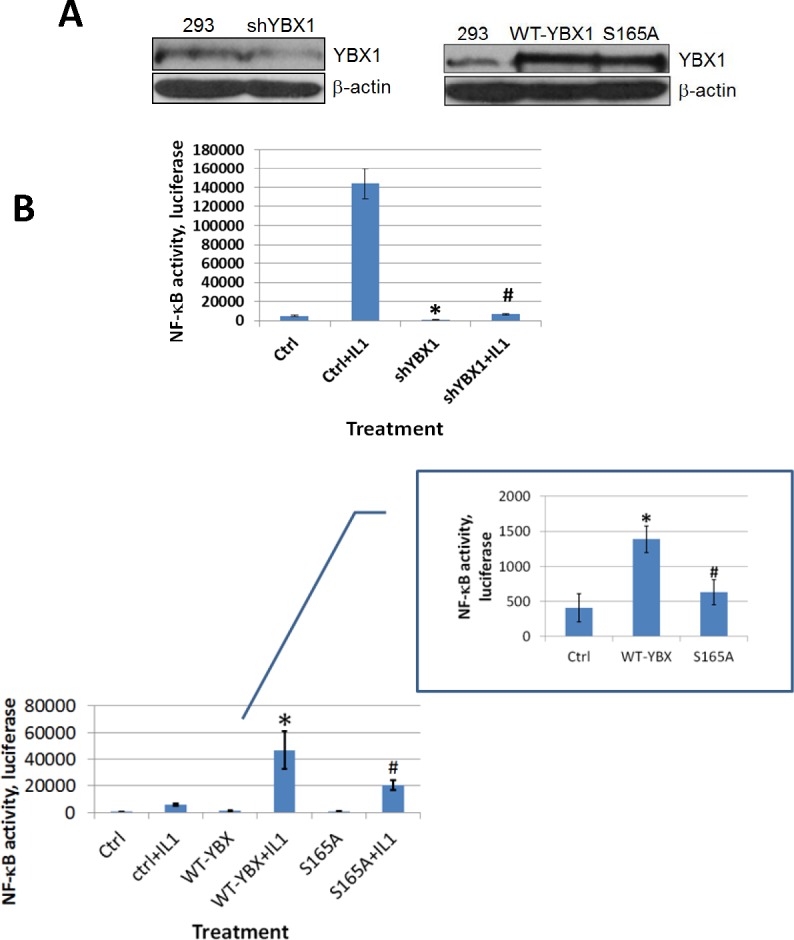

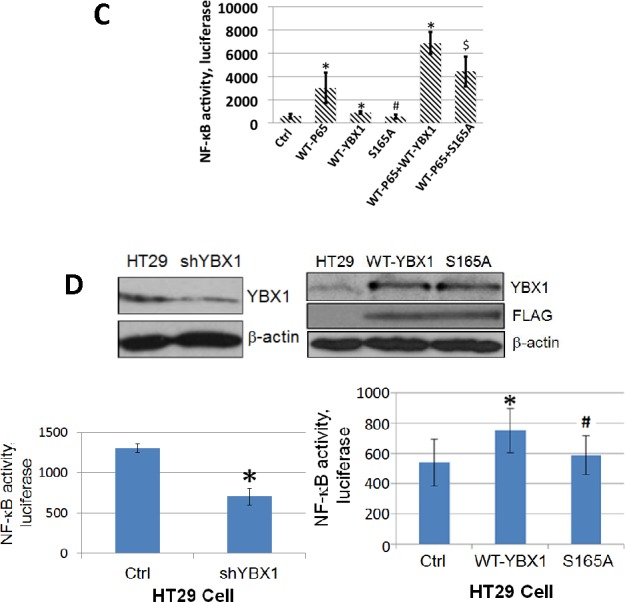
YBX1 is an activator of NF-κB and phosphorylation of S165 is critical for its NF-κB activating ability **A.** Western Blot, left panel, showing that YBX1 is knocked down by shRNAs. Right panel, showing that both wild type (WT-) YBX1 and mutant S165A-YBX1 were overexpressed at similar levels in 293 cells. **B.** κB-specific luciferase assay in 293 cells, top panel, NF-κB luciferase assay, showing that knockdown of YBX1 decreased IL-1β-induced NF-κB activation. The data represent the means ± SD from three independent experiments. *P < 0.05 *vs.* Ctrl group; #*P* < 0.05 *vs.* Ctrl+IL-1β group. Bottom panel inset, showing that overexpression of WT-YBX1 could activate NF-κB, while overexpression of S165A-YBX1 dramatically reduced NF-κB activation. Bottom panel main figure, showing that overexpression of WT-YBX1 could further enhance IL-1β-induced NF-κB activation, while overexpression of mutant S165A-YBX1 led to significantly lower NF-κB activation as compared to that of WT-YBX1. The data represent the means ± SD from three independent experiments. * *P* < 0.05 *vs.* Ctrl group; # *P* < 0.05 *vs.* WT-YBX1 group. **C.** κB-specific luciferase assay, showing that either WT-P65 or WT-YBX1 could activate NF-κB respectively. Additionally, WT-P65 and WT-YBX1 have synergistic effect on the activation of NF-κB. Mutant S165A-YBX1 showed much compromised ability for this synergistic effect. The data represent the means ± SD from three independent experiments. *P < 0.05 *vs.* Ctrl group. #*P* < 0.05 *vs.* WT-P65 group. $*P* < 0.05 *vs.* WT-P65/WT-YBX1 group. **D.** Western Blot in colon cancer HT29 cells, top left panel, showing that YBX1 was successfully knocked down by a pool of YBX1 specific shRNAs. Top right panel, showing that both WT-YBX1 and mutant S165A-YBX1 were overexpressed at similar levels in HT29 cells. Anti-Flag antibody was also used to show that both Flag-tagged WT-YBX1 and S165A-YBX1 was expressed at similar levels. Bottom left panel, showing that using shRNA to knock down YBX1 decreased NF-κB activity as compared to the HT29 control cells. The data represent the means ± SD for three independent experiments. *P < 0.01 *vs.* Ctrl group. Bottom right panel, κB-specific luciferase assay, showing that overexpression of mutant S165A-YBX1 led to decreased NF-κB activity as compared to that of WT-YBX1 cells. The data represent the means ± SD for three independent experiments. *P < 0.01 *vs.* Ctrl group; #*P* < 0.01 *vs.* WT-YBX1 group.

Although YBX1 is a known oncogene, to date no publications have shown that YBX1 could directly activate NF-κB in mammalian cells. Since NF-κB is frequently activated in cancer, we would like to examine whether YBX1 is an activator of NF-κB and whether phosphorylation of S165 plays a critical role in this process. In order to test this possibility, we used a pool of shRNAs to knockdown the expression of YBX1 protein in 293 cells (Figure 2A, left panel) or overexpressed either WT-YBX1 or S165A-YBX1 (Figure 2A, right panel). A NF-κB luciferase assay was carried out to determine the effect of YBX1 on NF-κB activation. As shown in Figure 2B, top panel, treating 293 cells with IL-1β greatly increased NF-κB activity, while knocking down the expression of YBX1 greatly reduced this effect, suggesting that YBX1 is a critical activator in IL-1β-induced NF-κB activation. Furthermore, overexpression of WT-YBX1 activated NF-κB, while overexpression of S165A-YBX1 mutant showed much reduced NF-κB activation as compared with WT-YBX1 (Figure 2B, bottom panel, inset). Impressively, this difference was further enhanced after three cell lines, 293 ctrl, WT-, and S165A-YBX1 cells, were further treated with IL-1β (Figure 2B, bottom panel, main figure), strongly suggesting that YBX1 is an activator of NF-κB, and phosphorylation of S165 plays a critical role in this function. Additionally, we overexpressed the WT-P65 subunit to activate NF-κB, a well-known phenomenon that has been previously reported [20]. As shown in Figure 2C, while sole overexpression of WT-YBX1 activated NF-κB, co-expression of both WT-YBX1 with WT-P65 led to synergic activation of NF-κB. In contrast, S165A-YBX1 had greatly reduced ability to activate NF-κB when expressed alone or together with WT-P65. Collectively, this data confirmed that S165 is important for NF-κB activation by YBX1 in 293 cells.

We previously reported that many types of cancer cells, including colon cancer HT29 cells, have constitutive NF-κB activity [20]. Since YBX1 is a tumor promoter, we wanted to examine whether YBX1 functions as a NF-κB activator in cancer cells. We used shRNA to knock down YBX1 (Figure 2D, top left panel) and we overexpressed either WT-YBX1 or S165A-YBX1 at similar levels in HT29 cells (Figure 2D, top right panel). A luciferase assay was carried out as described above. As shown in Figure 2D, knocking down YBX1 reduced the activity of NF-κB [bottom left panel], while overexpression of WT-YBX1 but not mutant S165A-YBX1 significantly increased the activity of NF-κB [bottom right panel]. Taken together, both the shRNA data and the overexpression data confirmed that YBX1 is an activator of NF-κB and S165 is critical for this activation in HT29 cells.

### Phosphorylation of YBX1 on S165 is critical for NF-κB regulated gene expression

**Figure 3:**
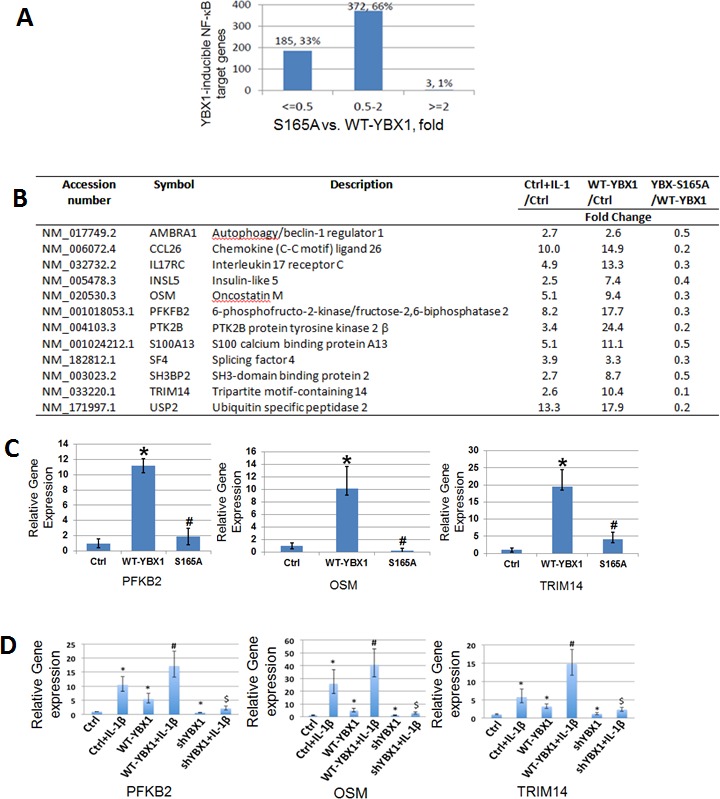
Phosphorylation of S165 is critical for the expression of a subgroup of NF-κB target genes **A.** Comparative analysis of YBX1-S165A mutant with WT-YBX1 overexpressing cells on YBX-inducible NF-κB target genes. **B.** A short list of typical NF-κB inducible genes that can be up regulated by WT-YBX1 but not by YBX1-S165A. **C.** Quantitative PCR (qPCR) analysis, showing confirmation of microarray data. Three genes: OSM, PFKB2, and TRIM14 show reduced gene expression in the mutant S165A-YBX1 as compared to that of WT-YBX1. The data represent the means ± SD from three independent experiments. *P < 0.05 *vs.* Ctrl group; #*P* < 0.05 *vs.* WT-YBX1 group. **D.** qPCR analysis, showing that PFKB2, OSM and TRIM14 genes were strongly induced upon IL-1β treatment. This effect was further augmented by the overexpression of WT-YBX1. However, the expression of these genes were greatly decreased upon shRNA knockdown of YBX1. The data represent the means ± SD for three independent experiments. *P < 0.01 *vs.* Ctrl group; #*P* < 0.01 *vs.* WT-YBX1 group; $*P* < 0.05 *vs.* WT-YBX1 group, with IL-1β treatment.

As post-translational modification of transcription factors is frequently related to differential gene expression [21], we examined how S165A mutation may affect NF-κB regulated gene expression. 293 cells with the overexpression of WT- or S165A-YBX1 were used to carry out Illumina microarray analysis. As shown in Figure 3A, compared with WT-YBX1, around 33% [185 genes] of NF-κB target genes were downregulated by two-fold or more in the cells expressing the S165A-YBX1 mutant protein. The remaining 66% of the genes were not significantly affected. A short list of representative genes that are downregulated by S165A-YBX1 is shown in Figure 3B. These genes include cytokines, chemokines, and signaling components that may be involved in tumorigenesis and metastasis, suggesting the important role that S165A-YBX1 may play in cancer. Several genes were further confirmed by performing qPCR analysis, including gene encoding the 6-phosphofructo-2-kinase [PFKB2], an enzyme that participates in fructose and mannose metabolism; gene encoding oncostatin M [OSM], a pleiotropic cytokine that belongs to the interleukin 6 group of cytokines; and gene encoding the tripartite motif-containing 14 [TRIM14], a member of TRIM proteins family that are involved in pathogen-recognition and regulation of transcriptional pathways in host defense. These genes showed reduced expression in the S165A-YBX1 mutant, as compared with the WT-YBX1 in 293 cells (Figure 3C), demonstrating that phosphorylation of YBX1 at S165 is critical for the activation of NF-κB-inducible genes. We further determined the effect of shRNA knockdown of YBX1 on the expression of these genes. As shown in Figure 3D, expression levels of PFKB2, OSM, and TRIM4 were strongly induced upon IL-1β treatment. This effect was further augmented by the overexpression of YBX1. However, using shRNA to knock down YBX1 significantly decreased the expression of these genes in the presence or absence of IL-1β treatment, confirming that YBX1 is important for the expression of these genes. Collectively, these data strongly supported the notion that phosphorylation of S165 on YBX1 plays an important role in NF-κB-inducible gene expression.

### Phosphorylation of S165 is critical for cell proliferation and tumor promoter activity in colon cancer HT29 cells

**Figure 4:**
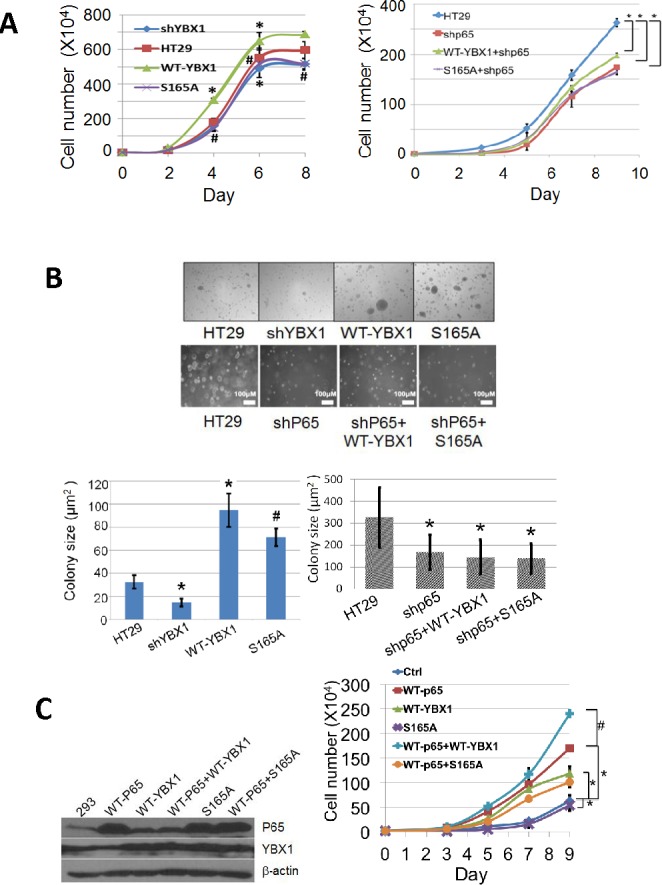
Phosphorylation of S165 of YBX1 is important for cell growth and colony independent growth in colon cancer HT29 cells **A.** Cell growth assay, left panel, showing that, compared to the HT29 control cells [symbol: solid square], overexpression of WT-YBX1 [symbol: solid triangle] promote cell growth, while overexpression of S165A-YBX1 [symbol: cross] or knockdown of YBX1 [symbol: solid diamond] failed to do so. The data represent the means ± SD from three independent experiments. *P < 0.05 *vs.* Ctrl group; #*P* < 0.05 *vs.* WT-YBX1 group. Right panel, showing that knockdown of P65 subunit of NF-κB decreased HT29 cell growth, while overexpression of WT-YBX1 [symbol: triangle] but not S165A-YBX1 [symbol, square] could partially rescue cell growth in shP65 knockdown cells. The data represent the means ± SD from three independent experiments. *P < 0.05 *vs.* Ctrl group. **B.** Soft agar assay, top row and bottom left panel, showing that overexpression of WT-YBX1 increases the size of colonies whereas knockdown of YBX1 showed the opposite effect in HT29 cells. Overexpression of S165A-YBX1 mutant showed decreased colony size as compared to that of WT-YBX1. Bottom row and bottom right panel, showing that knockdown of the P65 subunit of NF-κB decreased the size of colonies, while overexpression of either WT-YBX1 or S165-YBX1 could not rescue this phenotype. Bottom panel, quantification of colony number and colony size respectively for the soft agar assay. The data represent the means ± SD from three independent experiments. *P < 0.01 *vs.* Ctrl group. #*P* < 0.01 *vs.* WT-YBX1 group **C.** Left panel, Western assay, showing the expression levels of P65, WT-YBX1 or S165A-YBX1 after transient transfection in 293 cells. Right panel, cell growth was tested in the transfected cells. Overexpression of either P65 [symbol, square] or WT-YBX1 [symbol, triangle] but not S165A-YBX1 mutant [symbol, cross] significantly promoted cell growth. Co-overexpression of WT-P65 with WT-YBX1 [symbol, plus] but not S165A-YBX1 [symbol, circle] showed dramatically synergistic effect on cell growth. The data represent the means ± SD from three independent experiments. *P < 0.05 WT-P65 or WT-YBX1 or S165A *vs.* Ctrl group, # *P* < 0.01 WT-P65+WT-YBX1 group vs WT-P65.

In order to determine the effect of S165 phosphorylation on tumor promoter activity, cell growth was tested in colon cancer HT29 control, WT-YBX1, or S165A overexpressing stable cell lines. As shown in Figure 4A, left panel, knockdown of YBX1 greatly reduced cell growth, while overexpression of WT-YBX1 but not S165A-YBX1 mutant promoted cell growth, suggesting that YBX1 is critical for HT29 cell growth. To evaluate how YBX1 interplays with P65 in cell growth, we further knocked down the expression of P65 in the above group of cells. As shown in Figure 4A, right panel, cells grew significantly slower after P65 knockdown in all groups. Overexpression of neither WT-YBX1 nor S165-YBX1 could significantly rescue this phenotype, suggesting that YBX1 promoted cell growth via the interplay with P65. We further carried out soft agar assay for the groups of cells described above. Similarly, we observed that overexpression of WT-YBX1 but not S165A could dramatically promote colony formation. Using shRNAs to knockdown YBX1 greatly reduced colony formation (Figure 4B, top panel, top row, bottom left panel). Furthermore, knockdown of P65 with shRNA greatly diminished colony formation. Further overexpression of neither WT-YBX1 nor S165A could rescue this phenotype (Figure 4B, top panel, bottom row, bottom right panel). Collectively, the above findings suggest that S165 phosphorylation of YBX1 is critical for this function.

We also generated 293 cell lines overexpressing WT-P65 or WT-YBX1 or S165A-YBX1 (Figure 4C, left panel). Cell growth was further tested in these groups of cells. As shown in Figure 4C, right panel, overexpression of WT-P65 or WT-YBX1 but not the S165A mutant protein led to a significant increase in cell growth. Co-overexpression of WT-YBX1 but not S165A-YBX1 mutant with WT-P65 synergistically promoted cell growth. This data supported the notion that S165 phosphorylation of YBX1 is critical for promoting NF-κB-dependent cell growth by YBX1.

### YBX1 works downstream of IκBα

**Figure 5:**
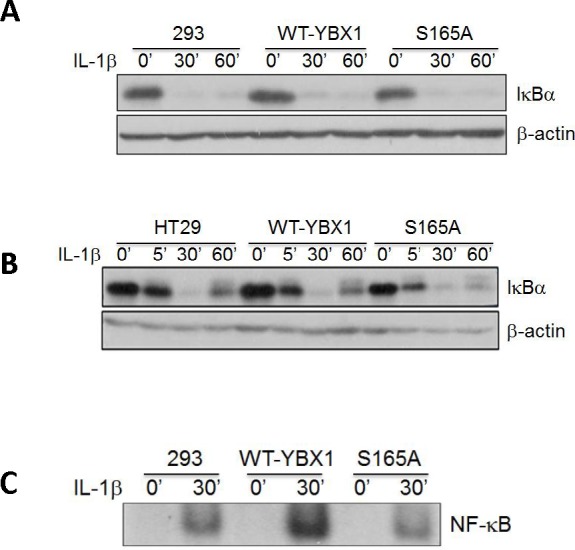

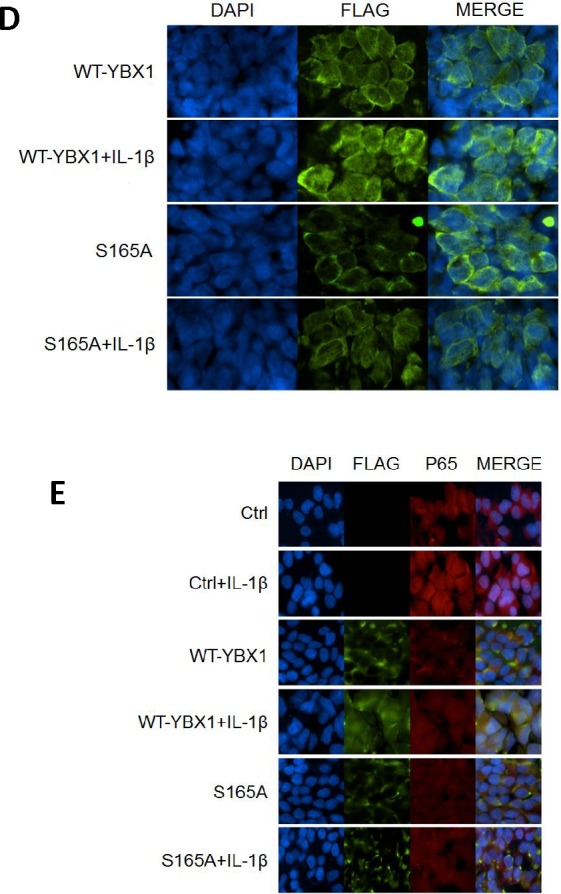
Activation of NF-κB by YBX1 functions downstream of IκBα degradation [A, B] Western Blot analyses, showing that overexpression of either WT-YBX1 or S165A-YBX1 did not affect the degradation of IκBα, suggesting that YBX1 functions downstream of IκBα degradation. **C.** EMSA assay, showing the overexpression of WT-YBX1 but not S165A-YBX1 enhanced NF-κB DNA binding ability upon IL-1β treatment, suggesting that phosphorylation of S165-YBX1 may enhance NF-κB DNA binding ability in nucleus. **D.** Immunofluorescence [IF] staining, showing the distribution of overexpressed WT-YBX1-Flag and S165A-YBX1-Flag in 293 cells. In untreated WT-YBX1 cells, YBX1 was located mainly in cytoplasm, but partly translocated into the nucleus after IL-1β treatment. However, YBX1 translocation was not observed in S165A-YBX1 mutant cells, before or after IL-1β treatment, suggesting that phosphorylation at S165 plays an important role in mediating the nuclear translocation of YBX1. DAPI was used for nuclear staining. **E.** IF experiment, showing that YBX1 co-localize with p65. IF experiment was carried out in 293, 293-WT-YBX1-Flag and S165A-YBX1-Flag stable cell lines. Anti-Flag was used to detect YBX1-Flag protein [green] and p65 antibody was used to detect endogenous P65 [red]. In 293 control cells, endogenous P65 is mainly located in cytoplasm while upon IL-1β treatment, P65 was observed in both cytoplasm and nucleus. No Flag-tagged YBX1 was observed in 293 cells. In WT-YBX1-Flag group, both Flag tagged YBX1 and endogenous p65 are located mainly in cytoplasm, but partly translocated into nuclear after IL-1β treatment and co-localized in nucleus. However, YBX translocation was not observed in S165A-YBX1-Flag cells, indicating that S165A eliminates YBX nuclear translocation ability. DAPI was used for nuclear staining.

IκBα degradation is a critical step in the activation of NF-κB triggered by cytokines such as IL-1β. Therefore, we treated either 293 (Figure 5A) or HT29 cells (Figure 5B) with the overexpression of WT-YBX1 or S165A-YBX1 mutant cells with IL-1β for different times to examine the effect of S165A-YBX1 on the degradation of IκBα as compared with that of WT-YBX1. As shown in Figure 5A, 5B, the NF-κB signaling pathway was activated by the degradation of IκB in a similar fashion in all the cell lines including control, WT-YBX1, and S165A-YBX1 upon IL-1β treatment. This result suggests that YBX1 may work downstream of IBα.

We then tested whether YBX1 affects NF-κB DNA binding ability. κB-specific EMSA analysis was carried out in 293 cells with overexpression of either WT-YBX1 or S165A-YBX1 proteins. As shown in Figure 5C, NF-κB DNA binding was greatly induced upon IL-1β treatment, confirming our previously reported finding [21]. Moreover, overexpression of WT-YBX1 but not S165A-YBX1 substantially enhanced IL-1β-induced κB binding ability, suggesting that S165 phosphorylation is critical for enhancing NF-κB DNA binding by YBX1 (Figure 5C).

How is YBX1 distributed in cells? Nuclear shuttling of YBX1 has been associated with its increased tumor promoter activity, and has been shown to occur upon stimulation with IL-1β [22]. In order to establish if phosphorylation at S165 is important for the nuclear shuttling of YBX1, we carried out an immunofluorescence [IF] experiment to check for Flag-tagged YBX1 localization. As visualized in Figure 5D, WT-YBX1 was mainly located in the cytoplasm, but partially translocated to the nucleus after IL-1β treatment. However, in S165A-YBX1 cells, S165A-YBX1 was mainly localized in the cytoplasm before and after IL-1β treatment, indicating that phosphorylation at S165 plays a critical role in mediating the nuclear translocation of YBX1.

We further carried out the IF experiments to examine the possibility of co-localization of WT-YBX1 and P65. 293, 293-WT-YBX1 and S165A stable cell lines were used as models. Flag antibody was used to detect Flag-tagged YBX1 [green], and P65 antibody [red] was used to detect endogenous p65. DAPI was used for nuclear staining. As observed in Figure 5E, endogenous p65 was mainly located in cytoplasm in non-stimulated 293 control cells. Upon IL-1β treatment, P65 was redistributed in both cytoplasm and nucleus. As expected, no Flag-tagged YBX1 was observed in 293 control cells. In WT-YBX1 overexpressing cells, both Flag-tagged YBX1 and endogenous P65 were mainly located in the cytoplasm, but partially translocated and co-localized in the nucleus after IL-1β treatment. On the other hand, YBX1 translocation was not observed in S165A-YBX1 cell lines in the absence or presence of IL-1β treatment, indicating that S165A disrupts the nuclear co-localization of YBX1 and P65 in 293 cells.

### Expression of YBX1 increases with colon cancer progression

**Figure 6:**
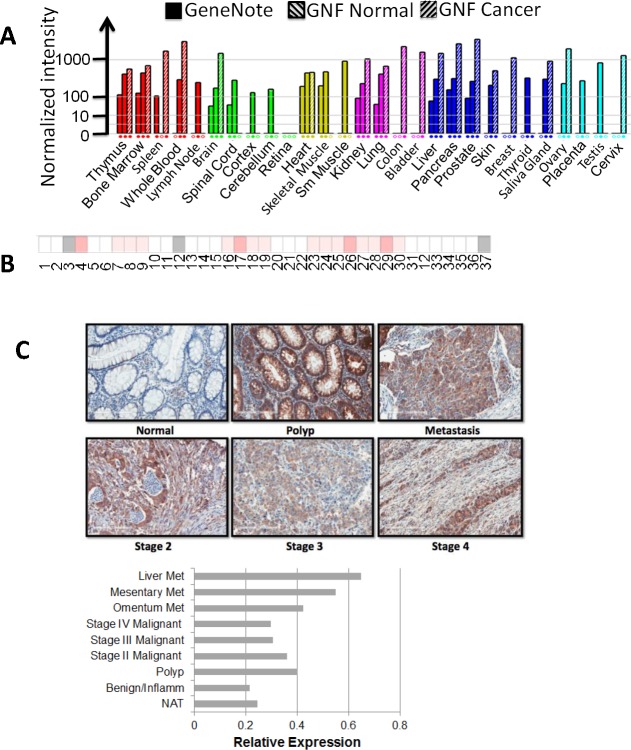
YBX1 is overexpressed in many types of cancer, especially in colon cancer **A.** GeneNote data, showing the expression level of YBX1 in different types of cancers; colon cancer shows very high expression level of YBX1. **B.** Oncomine data, showing the comparison of the expression of YBX1 in 37 colon cancer *vs.* normal datasets. The intensity of red color corresponds to the level of expression of YBX1, indicating that YBX1 is overexpressed in the specimens from many colon cancer patients. **C.**. Tissue microarray assay of YBX1, top panel, showing tissue sections stained with anti-YBX1 antibody, demonstrating elevated levels of YBX1 staining in polyps and different stages of colon cancer as compared to the normal tissue. Magnification fold: 20X. Bottom panel, showing relative expression of YBX1 across different stages of colon samples, polys and tumors showed relatively higher levels of YBX1 expression than that of normal adjacent tissue. Abbreviations: Met, metastasis; NAT, normal adjacent tissue.

As YBX1 is a known oncogene, we further examined the expression levels of YBX1 in different types of cancers. GeneNote data (Figure 6A) indicated that the expression level of YBX1 is increased in the cancers of the thymus, bone marrow, spleen, whole blood, brain, kidney, lung, colon, bladder, liver, pancreas, prostate, skin, breast, salivary gland, ovary, and cervix, with strikingly elevated levels of YBX1 in colon cancer. Oncomine data also indicated that out of the 37 colon cancer patient specimens, the majority showed increased expression of YBX1 (Figure 6B). We further carried out tissue microarray [TMA] analysis [US Biomax Inc., Rockville, MD] for YBX1 in specimens from both normal and different stages of colon cancer by using immunohistochemical [IHC] methods. Compared with normal specimens, a marked increase of YBX1 expression was observed in progressive stages of colon cancer (Figure 6C). Interestingly, a dramatic increase of YBX1 expression was also observed in tissue samples isolated from polyps compared with the normal specimens. Overall, this data strongly suggested that YBX1 potentially plays a very important role in colon cancer development, and possibly in the transition between polyps and cancerous cells as well.

### Hypothetical model

**Figure 7:**
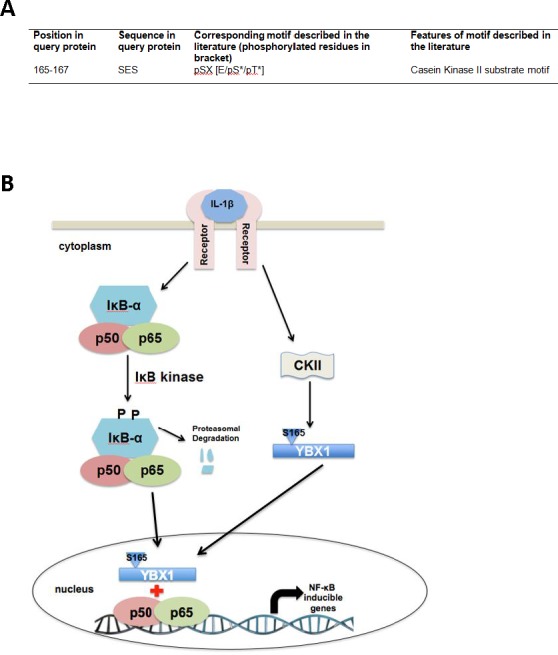
Hypothetical Model **A.** Casein kinase II is predicted to be the kinase that is responsible for the phosphorylation of S165 of YBX1. **B.** Model, hypothesizing that in the presence of external stimuli like IL-1β, the IκB kinase phosphorylates IκBα. This leads to the degradation of IκB. The free P65/P50 heterodimer then migrate to the nucleus to bind to κB-binding sites on the promoters of specific genes, leading to their activation. On the other hand, IL-1β has also been shown to stimulate YBX1 activity. We hypothesize that stimulation with IL-1β activates CKII, which then promotes phosphorylation of YBX1 on S165 which then leads to increased activation of NF-κB, possibly by increasing the DNA binding ability of NF-κB, and enhancing the expression of NF-κB-inducible genes, thus promoting cancer progression.

By using human protein research domain software, we predicted that S165 of YBX1 is phosphorylated by casein kinase II [CKII] (Figure 7A). Collectively, based on all the data we have presented, we hypothesize that in the presence of an NF-κB activating cytokine, such as IL-1β, the IκB kinase phosphorylates IκBα, which leads to the degradation of IκB. The free P65/P50 heterodimer then migrates to the nucleus to bind to κB binding sites on the promoters of specific genes, leading to their activation. Meanwhile, IL-1β leads to the activation of CKII which then phosphorylates YBX1 on S165. This event further activates NF-κB possibly by increasing the translocation of NF-κB into the nucleus and enhancing NF-κB DNA binding ability, thereby promoting NF-κB-inducible gene expression (Figure 7B).

## DISCUSSION

Aberrant activation of the NF-κB signaling pathway is associated with most cancers [15]. NF-κB regulates expression of genes involved in the cell cycle, apoptosis, immune response, and tumorigenesis [14]. Thus, targeting this pathway holds tremendous therapeutic potential in treating cancer and other inflammatory diseases. It is also now a widely accepted view that YBX1 is an oncoprotein that promotes the most of the famous ‘hallmarks of cancer’ proposed by Hanahan and Weinberg [6]. However, to date, there are no reports regarding the direct crosstalk between NF-κB and YBX1.

In this study, we showed that overexpression of WT-YBX1 could activate NF-κB, and this effect is dramatically enhanced when cells were further treated with NF-κB activating cytokines such as IL-1β. Moreover, using mass spectrometry we discovered a previously unknown phosphorylation of Ser165 on YBX1. We proved that mutation of S165 to “A” greatly reduced the NF-κB activating ability of YBX1, and therefore, greatly reduced cell growth and colony formation in colon cancer HT29 cells.

Sutherland and colleagues found that S102 of YBX1 is phosphorylated upon treatment with IGF1. Mutation of the S102 site of YBX1 inhibited tumor growth via the Akt/PKB pathway in breast cancer [10]. It is interesting that we did not observe phosphorylation of S102 in IL-1β treated cells by either mass spectrometry or western analysis, suggesting that different cytokines could lead to differential phosphorylation in cancer cells. Different from IGF1, IL-1β is a very classical NF-κB activating cytokine, which is frequently found in the tumor environment. The identification of S165 phosphorylation by IL-1β expands our current knowledge and provides deeper insight into the complicated phosphorylation pattern of YBX1.

Moreover, we reported the interesting and important connection between YBX1 modification and NF-κB activation. To our knowledge, this is the first report to suggest that YBX1 is a direct activator of NF-κB, and S165 phosphorylation is critical for its NF-κB activating function.

Importantly, GeneNote data, Oncomine data, together with our tissue microarray data (Figure 6) strongly support the overexpression of YBX1 in cancer, particularly, in colon cancer. Our tissue microarray data suggested that YBX1 is overexpressed not only in different stages of colon cancer specimens but also in polyps, indicating a potential role YBX1 may play in early stage of colon cancer development.

By using the Human Protein Reference Database, we predicted that CKII may be the kinase to phosphorylate YBX1 on S165. CKII is an important kinase that has been linked to the activation of NF-κB by reactive oxidase species [ROS] [23] as well as to the NF-κB activation in pancreatic β cells and cancers such as glioblastoma [24, 25]. In addition, CKII was reported to be involved in cell cycle control and DNA repair and has been shown to activate the Wnt signaling pathway. In cancer, Wnt has been implicated in promoting the epithelial-mesenchymal transition that contributes to the development of the cancer phenotype [26]. YBX1 has been shown to bind to the promoters of a number of Wnt pathway proteins. Hence, further research involving validating the role of CKII in our model could help shed light on the mechanism underlying S165 phosphorylation of YBX1 and help to better understand the crosstalk between different signaling pathways that are linked through the activation of YBX1 by S165 phosphorylation.

Our current study has great significance by linking two well-known cancer culprits: NF-κB and YBX1. Although targeting NF-κB is critical to cancer therapy, due to its critical role in normal cellular functions, it is generally not a wise strategy to directly target NF-κB in cancer. For this reason, targeting pathway-specific regulators of NF-κB would seem to be a more favorable approach in this regard.

Our current study emphasizes that phosphorylation of S165 on YBX1 is critical for the regulation of a subgroup of NF-κB target genes; therefore, modulation of this activity by blocking S165 phosphorylation of YBX1 could be a promising approach for inhibiting tumor growth. Furthermore, although our study is mainly focused on colon cancer, it is highly possible that phosphorylation of S165 of YBX1 also plays an important role in other types of cancer as well. This would also be a potential new aspect we would like to explore in the future.

## MATERIALS AND METHODS

### Cell lines and antibodies

The 293 cell line has been described previously [19]. The HT29 colon cancer cell line was purchased from the American Tissue Culture Collection [ATCC, Manassas, VA, USA] and was cultivated in RPMI-1640 media with 100 U/ml penicillin, 100 μg/ml streptomycin, and 10% fetal calf serum. The following antibodies were obtained from commercial sources: anti-YBX1 [Abcam, Cambridge, MA, USA], anti-NF-κB p65 [Santa Cruz Biotechnology Inc. Dallas, Texas, USA], anti-IκBα [Santa Cruz Biotechnology Inc. Dallas, Texas, USA], and anti-β-actin [Sigma-Aldrich, St. Louis, MO, USA].

### Construction of stable overexpressing WT-YBX1, mutant S165A-YBX1, and shYBX1 in HT29 cells

Flag-tagged WT-YBX1 cDNA was amplified by reverse transcription from total mRNA derived from 293 cells. Sequence was confirmed and then cloned into pLVX-IRES-puro vector [19]. Mutation of S165 residue to A was generated using QuikChange II XL Site-Directed Mutagenesis Kit following manufacturer's protocol (Agilent Technologies, Inc., Santa Clara, CA). The mutated site was then confirmed by sequencing. The shRNA pool against YBX1 was purchased from Sigma-Aldrich, St. Louis, MO, USA. All cell lines were generated using either 293 or HT29 colon cancer cell lines. To generate the stable cell lines, the lentiviral plasmid containing the DNA of interest or shRNAs were transected into a 293T packaging cell line to produce viruses which then were collected and used to infect 293 or HT29 cells. 48 h after infection, cells were further selected with 1μg/mL of puromycin, since the lentiviral vector construct was comprised of a puromycin resistance gene. Expression of the respective constructs was confirmed with Western Blotting using specific antibodies.

### Transfections and luciferase assays

Constructs were transfected into cell lines using the Lipofectamine and PLUS Reagents [Life Technologies Invitrogen, Grand Island, NY]. For NF-κB luciferase assays, the κB-luciferase construct p5XIP10 κB [20] was transfected transiently into the cells, and luciferase activity was assayed 48 h later. A β-galactosidase construct was co-transfected to normalize for transfection efficiency. Transfections and luciferase assays were carried out as previously described [21].

### Western analyses

Cells were cultured to ∼95% confluence, and samples were collected and assayed by the Western method as described previously [20]. Different antibodies were used based on different experiments described in the text.

### Electrophoresis gel mobility assay [EMSA]

The oligomer used for the NF-κB binding site was 5′AGTTGAGGGGACTTTCCCAGGC-3′ [Santa Cruz Biotechnology, Inc. Dallas, TX]. It was labeled with [γ-^32^P] ATP by the polynucleotide kinase method, following the protocol provided by Promega Corporation [Madison, WI]. Whole cell lysates were prepared and analyzed essentially as previously described [20].

### Cell growth and soft agar assays

HT29 cells overexpressing WT-YBX1, S165A-YBX1, and shRNA-YBX1 knockdown cell lines were plated at 2 × 10^4^ cells/well in a six-well plate with 3ml RPMI-1640 media. Cells were seeded in triplicates and counted at different time points *i.e.* day 2, 4, 6, 8 using a cell counting chamber. For soft agar assays, type VII agarose [Sigma] was autoclaved and mixed with RPMI-1640 cell growth medium. Cell culture dishes were coated with 1.2% type VII agarose as the bottom layer. Cells were resuspended in 0.6% of type VII agarose, and plated on top of the bottom layer. Cells were cultured for 2-3 weeks before being checked under a microscope, measured and quantified with the aid of ImageJ software [http://imagej.nih.gov/ij/].

### Mass spectrometry [MS] experiments

Ten 15-cm plates of 293C6 cells with the stably expressed WT-YBX1-Flag protein were cultured to 80% confluence. Five were used as control, and the other 5 plates were treated with IL-1β for 1 h. Cells were then lysed with coimmunoprecipitation buffer [1% Triton X-100, 50 mM Tris-HCl, pH 7.4, 150 mM NaCl, 1 mM EDTA, 1 mM sodium orthovanadate, 20 μM aprotinin, and 1 mM phenylmethanesulfonyl fluoride and pepstatin A]. After spinning the debris for 10 min at 4°C, the supernatant solution was incubated with EZview Red anti-Flag M2 affinity gel overnight at 4°C. Gel beads were washed with 20 volumes of Immunoprecipitation buffer [21], with rotation at 4°C for 5 min each time. Protein was eluted with Flag peptide [Sigma], following Sigma's standard protocol. The supernatant solution was mixed with 5XSDS sample loading buffer, boiled for 5 min, and separated in a 10% Tris-HCl SDS/PAGE gel [21]. The gel was then treated with fixing buffer [50% ethanol, 10% acetic acid] for 20 min and washed with distilled water for 1 h before being stained with GelCode Blue stain [Pierce] overnight. The gel was destained with distilled water for 2 h before analysis by MS.

### Protein in-gel digestion

Gel pieces cut from SDS/PAGE gels were excised and subjected to in-gel tryptic digestion. The excised bands were washed twice with 100 mM ammonium bicarbonate containing 50% acetonitrile for 1 h and twice with acetonitrile for 10 min. The proteins in the gels were then treated with 20 mM Dithiothreitol [DTT] at room temperature for 30 min followed by 50 mM iodoacetamide for 30min in 100mM ammonium bicarbonate. After the treatment, the reagents were removed, and the gel pieces were washed with 100 mM ammonium bicarbonate and then dehydrated in acetonitrile. The dried gel pieces were then rehydrated in 50 mM ammonium bicarbonate containing sequencing grade modified trypsin for overnight digestion. Tryptic peptides were extracted from the gel with 50% acetonitrile in 5% formic acid.

### Tandem mass spectrum analysis

The proteolytic digests were analyzed using a LTQ Orbitrap XL linear ion trap mass spectrometer [Thermo Fisher Scientific] coupled with Ultimate 3000 HPLC system [Dionex]. The reverse-phase C18 column [0.075 × 150 mm, Dionex] was equilibrated with 0.1% formic acid 4% acetonitrile [vol/vol], and the proteolytic digests were injected on it. A linear gradient of acetonitrile from 4% to 40% in water in the presence of 0.1% formic acid over a period of 45 min was used at a flow rate of 300 nL/min. The spectra were acquired by data-dependent methods consisting of a full scan [m/z 400-2,000] and then tandem MS on the five most abundant precursor ions. The previously selected precursor ions were scanned once during 30 s and then were excluded for 30 s. The obtained data were submitted to Mascot software [Matrix Science] to search for phosphorylated residues on the YBX1 protein. The tandem mass spectra of the possibly modified peptides were further interpreted manually.

### Illumina microarrays

RNA [250 ng] was reverse-transcribed into cDNA and labeled with biotin-UTP using the Illumina TotalPrep RNA Amplification Kit [Ambion/Applied Biosystems]. The amount of cDNA was determined by using a nanodrop spectrophotometer, and the cDNA quality [size distribution] was further analyzed in a 1% [wt/vol] agarose gel. cDNA was hybridized to Illumina Human Ref-v3 v1 Expression BeadChips and scanned in a BeadArray Reader using standard protocols [provided by Illumina]. Illumina's BeadStudio software was used for data analysis.

### Immunofluorescence [IF] experiment

Coverslips were coated with 0.1% sterile gelatin for 2 h and dried for 30min at R.T. 1×10^5^ cells were then seeded onto coverslip per well in a 24-well plate. After overnight culture, cells were treated with or without IL-1β for 1h to continue with IF experiments. Cells were fixed with 4% of formaldehyde for 30min and then blocked with blocking buffer for 10min at R.T. Coverslips were further probed with anti-Flag antibody for flag-tagged WT-YBX1 or S165A and Alexa Fluor 488 [green] goat anti-mouse IgG [Life Technologies]. For double staining, anti-Flag antibody for Flag-tagged WT-YBX1 or S165A and anti-p65 for p65 were used then further probed with Alexa Fluor 488 [green] goat anti-mouse IgG and Alexa Fluor 680 [red] goat anti-Mouse IgG [Life Technologies], respectively. Before sealing the coverslips, mounting media with DAPI was used to stain the nucleus. The slides were examined under Nikon Eclipse 80i with 60x magnification.

### Immunohistochemistry [IHC] experiment

Colon cancer tissue microarray comprising matched normal adjacent and various clinical stages of colon carcinoma tissue was obtained from US Biomax Inc. [Rockville, MD]. Hematoxylin-eosin [H&E] was performed using routine methods. The tissue microarray were blocked with protein blocking solution [Dako Corp.] for 30 min. All subsequent staining steps were performed using the Dako FLEX SYSTEM on an automated Immunostainer. Incubations were done at room temperature and Tris buffered saline plus 0.05% Tween 20, pH 7.4 [Dako Corp] was used for all washes and diluents. Thorough washing was performed after each incubation. Anti-YBX1 primary antibody was used to detect YBX1 localization. Horseradish peroxidase conjugated secondary antibody was then used, followed by addition of the chromogen, which produced a brown precipitate at the site of secondary antibody binding. The Aperio whole slide digital imaging system was used for imaging. The system imaged all slides at 20X.

### Statistical analysis

Statistical analysis was performed using Prism 6 software [GraphPad, San Diego, CA]. The data represent the mean ± SD from three independent experiments. A two-tailed Student's t test was used while comparing two means and to test for significant differences between relative luciferase activity and relative gene expression in different groups. All statistics were calculated on triplicate experiments. For all statistics, *P* < 0.05 was considered to be statistically significant.
